# Impact of Dietary Cholesterol on the Pathophysiology of Infectious and Autoimmune Disease

**DOI:** 10.3390/nu10060764

**Published:** 2018-06-13

**Authors:** Catherine J. Andersen

**Affiliations:** Department of Biology, Fairfield University, Fairfield, CT 06824, USA; candersen@fairfield.edu; Tel.: +1-203-254-4000

**Keywords:** dietary cholesterol, immunity, infectious disease, autoimmune disease, inflammation, lipoprotein metabolism, eggs

## Abstract

Cellular cholesterol metabolism, lipid raft formation, and lipoprotein interactions contribute to the regulation of immune-mediated inflammation and response to pathogens. Lipid pathways have been implicated in the pathogenesis of bacterial and viral infections, whereas altered lipid metabolism may contribute to immune dysfunction in autoimmune diseases, such as systemic lupus erythematosus, multiple sclerosis, and rheumatoid arthritis. Interestingly, dietary cholesterol may exert protective or detrimental effects on risk, progression, and treatment of different infectious and autoimmune diseases, although current findings suggest that these effects are variable across populations and different diseases. Research evaluating the effects of dietary cholesterol, often provided by eggs or as a component of Western-style diets, demonstrates that cholesterol-rich dietary patterns affect markers of immune inflammation and cellular cholesterol metabolism, while additionally modulating lipoprotein profiles and functional properties of HDL. Further, cholesterol-rich diets appear to differentially impact immunomodulatory lipid pathways across human populations of variable metabolic status, suggesting that these complex mechanisms may underlie the relationship between dietary cholesterol and immunity. Given the Dietary Guidelines for Americans 2015–2020 revision to no longer include limitations on dietary cholesterol, evaluation of dietary cholesterol recommendations beyond the context of cardiovascular disease risk is particularly timely. This review provides a comprehensive and comparative analysis of significant and controversial studies on the role of dietary cholesterol and lipid metabolism in the pathophysiology of infectious disease and autoimmune disorders, highlighting the need for further investigation in this developing area of research.

## 1. Introduction

Regulation of the immune system is essential to the maintenance of health and protection from acute and chronic diseases [1,2,3]. The initial response to pathogens or tissue injury involves activation of innate immune system cells, including antigen-presenting macrophages and dendritic cells, monocytes, granulocytes (neutrophils, basophils, eosinophils), and natural killer cells [4]. Exogenous microbes and endogenous stimuli released from injured host cells serve as pathogen- (PAMPs) or damage-associated molecular patterns (DAMPs), respectively, which directly bind and stimulate cognate pattern-recognition receptors, initiating cascades of pro-inflammatory signaling and immune cell activation [5,6]. Coordinated efforts of antigen-presenting cells lead to selective activation of adaptive immune cells, including B and T lymphocytes, conferring cell- and antibody-mediated immunity [7]. Immune responses lead to extensive proliferation of leukocytes and the generation of cytotoxic and pro-inflammatory mediators that allow for destruction and clearance of the initial pathogenic stimuli [4]. Resolution of the immune response involves suppression of pro-inflammatory cytokines and interleukins, and concomitant increases in anti-inflammatory compounds that promote tissue healing and return to homeostasis [8].

Disordered immune responses often stem from (1) a failure of immune cells to appropriately recognize and respond to pathogenic factors; (2) inappropriate recognition and response to self-tissue (i.e., in the case of autoimmunity); and (3) failure of pro-inflammatory immune responses to adequately resolve, leading to prolonged, chronic inflammation [8,9,10]. Dysregulation of the immune system has been implicated in a broad range of acute and chronic conditions, ranging from increased susceptibility to infection, autoimmunity, and chronic inflammatory diseases, such as asthma, osteoarthritis, cardiovascular disease, non-alcoholic fatty liver disease, and cancer [11,12,13,14,15,16,17]. Thus, identifying strategies and interventions aimed at optimizing appropriate immune responses and leukocyte activity may minimize the risk of acute and chronic diseases. 

Research has demonstrated that various dietary patterns and bioactives have the capacity to modulate innate and adaptive immune responses, as well as immune-mediated inflammation [18,19,20,21,22,23]. While most extensively evaluated within the context of cardiovascular disease, dietary cholesterol may serve as an important regulator of immune cell activity and inflammation, with implications for risk and treatment of infectious disease and chronic autoimmune disorders [24,25,26,27,28]. Understanding the relationship between dietary cholesterol and immunity is particularly timely, given the removal of cholesterol intake limitations in the most recent Dietary Guidelines for Americans 2015–2020, as well as the American Heart Association/American College of Cardiology 2013 lifestyle recommendations aimed at reducing cardiovascular disease risk [29,30]. This review discusses the role of cholesterol and lipid metabolism in regulating leukocyte activity, highlighting research that elucidates the impact of dietary cholesterol on immune function beyond cardiovascular disease. 

## 2. Role of Cholesterol in Immunity

Cellular and systemic cholesterol metabolism play a significant role in the regulation of immune cell activity [25]. The majority of cellular cholesterol is located within the plasma membrane, particularly within cholesterol- and sphingomyelin-rich lipid raft regions [31]. Lipid rafts provide essential structure and scaffolding to allow for recruitment and signaling of immune receptors [23,32]. Cellular cholesterol depletion has been shown to disrupt raft stability and formation, as well as suppress toll-like receptor 4 (TLR4)- and T cell receptor (TCR)-mediated immune activation and pro-inflammatory signaling, whereas these processes are enhanced by cholesterol loading [33,34,35,36,37]. Lipid raft content and inflammatory activation of leukocytes may be further modulated through interactions with lipoproteins, which mediate cholesterol loading and efflux, serve as carriers of immunomodulating proteins, and directly interact with, and sequester, pathogenic stimuli [25]. Markers of cellular cholesterol metabolism and lipoprotein function have been found to be altered in models of infectious and chronic diseases, whereas lipid-lowering therapies have been shown to exhibit variable potential in the treatment of immune disorders [38]. The following sections highlight the role of lipid rafts and lipoprotein interactions in immunity.

### 2.1. Role of Cholesterol in the Pathophysiology of Infectious Disease

#### 2.1.1. Role of Lipid Rafts and Lipoprotein Interactions in Bacterial Pathogenesis

In addition to regulating cellular signaling and inflammatory potential, lipid rafts serve as a point of pathogen association and colonization [39]. Membrane cholesterol within lipid rafts has been implicated in the pathology of infection by numerous bacterial strains, including *Camylobacter jejuni* and *Aggregatibacter actinomycetemcomitan*, which cause enterocolitis and periodontal disease, respectively [40,41,42]. Cholesterol is thought to mediate host cell infection by these Gram-negative bacterial strains through recognition and binding of the virulence factor cytolethal distending toxin (CDT), and mediating subsequent translocation of active toxin subunits [40,41]. *Helicobacter pylori* infection, which is associated with gastric inflammation, ulcers, and cancer, is additionally mediated by interactions between membrane cholesterol and the virulence protein cytotoxin-associated gene A (CagA), whereas cellular cholesterol depletion by methyl-β-cyclodextrin (MβCD) or lovastatin was shown to reduce CagA translocation and pro-inflammatory interleukin 8 (IL-8) secretion in human gastric adenocarcinoma AGS cells [43,44]. Cellular cholesterol content of host cells has also been implicated in infection by multiple *Chlamydia* species associated with urogenital tract infections and respiratory pneumonia (*C. trachomatis* and *C. pneumonia*)[45,46], tick-borne pathogens *Anaplasma phagocytophilium* and *Ehrlichia chaffeensis* [47], as MβCD-mediated depletion of membrane cholesterol impairs various stages of bacterial infection.

Lipoproteins and their associated transporters additionally impact immune function and disease pathogenesis. Deletion of macrophage-specific ATP-binding cassette transporter A 1 (ABCA1), which effluxes cholesterol and phospholipids to apolipoprotein A 1 (apoA1) to form HDL, is associated with increased lipid raft formation and hyper-responsiveness to TLR2, TLR4, TLR7, and TLR9 ligands [48]. Myeloid cell-specific deletion of ABCA1 has additionally been shown to promote enhanced inflammatory immune responses and clearance of *Listeria monocytogenes* in mice [34], whereas upregulation of HDL pathways may improve response to bacterial pathogens in other models. In addition to mediating cholesterol efflux, HDL and other lipoproteins can bind and neutralize Gram-negative bacteria-derived lipopolysaccharide (LPS) and Gram-positive bacteria-derived lipoteichoic acid (LTA), thereby suppressing inflammatory immune responses and promoting pathogen clearance [49,50,51]. Accordingly, higher levels of serum HDL-cholesterol, reconstituted HDL infusion, and apoA1 overexpression have been shown to improve clinical outcomes of sepsis [52,53,54,55].

It is also important to note that bacterial infections disrupt cellular and systemic lipid metabolism. *C. pneumonia* infection has been shown to increase LDL uptake, suppress expression of ABCA1 and ABCG1, and reduce cholesterol efflux to apoA1, leading to greater cholesterol loading of host cells that promotes bacterial propagation [56,57]. Plasma cholesterol levels—particularly HDL-cholesterol—are rapidly reduced in severe sepsis, and HDL particle composition is altered to contain greater levels of pro-inflammatory serum amyloid A (SAA), and reduced apoA1, apoM and sphingosine-1-phosphate (S1P), thus impairing the cholesterol efflux and anti-inflammatory properties of HDL [38,58,59,60].

#### 2.1.2. Role of Lipid Rafts and Lipoprotein Interactions in Viral Infection

In addition to bacterial pathogenesis, membrane cholesterol within lipid rafts plays an important role in viral infection. Danthi and Chow [61] demonstrated that MβCD-mediated membrane cholesterol depletion inhibited uptake of poliovirus, whereas repletion of cellular cholesterol partially restored viral uptake levels. Influenza viruses that encode hemagglutinin and neuraminidase have similarly been shown to associate with lipid rafts [62], whereas statin treatment has been shown to have both beneficial and neutral effects on reducing influenza-related morbidity and mortality [63,64,65]. Interestingly, statin usage reduces influenza vaccine efficacy against influenza A (H3N2), but not the pandemic influenza A (H1N1) or influenza B viral strains [66]. These findings suggest that anti-inflammatory properties of statins may lead to suppressive immune responses that impact strain-specific viral immunity [67,68].

Similar to mechanisms of bacterial pathogenesis, HDL and its associated efflux transporters have been implicated in human immunodeficiency virus (HIV) and hepatitis C virus (HCV) infection [69,70]. HIV infection has been shown to suppress ABCA1-mediated efflux via the viral accessory protein Nef, promoting viral replication [69,71]. Accordingly, upregulation of ABCA1 by liver X receptor (LXR) activation and all-*trans* retinoic acid reduces T lymphocyte cholesterol content and inhibits HIV replication [72,73]. Conversely, HDL-mediated pathways may promote HCV infection [38]. HCV can be carried and sequestered by HDL and other lipoproteins, protecting the virus from neutralizing antibodies [70]. Cellular uptake of HCV is thought to be mediated by a variety of receptors—including the LDL-receptor and scavenger receptor class B type I (SR-BI), the latter of which facilitates bidirectional cholesterol exchange with HDL [74,75,76]. Thus, HDL may play opposing roles in the inhibition or promotion of different viral infections.

Interestingly, in a recent study assessing 106,553 individuals from the Copenhagen General Population and Copenhagen City Heart studies, both low (<31 mg/dL) and very high (>100 mg/dL) HDL-cholesterol levels were shown to be associated with an increased risk of infectious disease [77]. Low HDL-cholesterol appeared to have a greater impact on infectious disease risk, with a 75% increased risk of disease, vs. a 43% increased risk in individuals with very high HDL-cholesterol [77]. In line with the findings outlined above, it is plausible that elevated HDL may promote infection by certain pathogens, or that elevated HDL-cholesterol levels may be indicative of impaired HDL clearance via reverse cholesterol transport or greater levels of dysfunctional HDL [38,70,78,79].

### 2.2. Role of Cholesterol in the Pathophysiology of Autoimmune Disease

In addition to infectious disease pathways, cholesterol metabolism appears to play an important role in autoimmune disease. In patients with systemic lupus erythematous (SLE), T lymphocytes exhibit greater degrees of lipid raft formation and altered raft composition, leading to increased TCR-mediated signaling and inflammation [80,81]. Statins have been shown to mitigate inflammatory dysfunction in animal and ex vivo cell models of lupus [82,83], but have not been found to improve SLE Disease Activity Index (SLEDAI) scores—a measure of clinical disease status [84]. Activated T cell populations have additionally been observed in synovial joint fluid from rheumatoid arthritis (RA) patients, whereas conflicting studies have found that statin treatment may decrease or increase risk for developing RA [85,86,87,88]. 

In contrast to hyperlipidemic trends in certain autoimmune conditions, metabolomic analysis has shown that cholesterol levels in bronchoalveolar lavage fluid are reduced in ovalalbumin-sensitized BALB/c mouse models of asthma, whereas cholesterol content is normalized by dexamethasone treatment [89]. Pulmonary cholesterol levels were negatively correlated with numbers of macrophages, eosinophils, neutrophils, and lymphocytes, in contrast to research on other disorders suggesting that cholesterol loading promotes leukocytosis of pro-inflammatory cell types [36,89]. Fessler et al. [90] additionally found that serum total cholesterol and non-HDL-cholesterol levels were lower in asthma patients in the 2005–2006 National Health and Examination Survey dataset. Peng and Huang [91] found age-specific associations in a recent meta-analysis, in that HDL-cholesterol levels were reduced in children in asthma, whereas LDL-cholesterol levels were elevated in asthmatic adults. From these findings, it is unclear whether altered cholesterol levels are a result of asthma, or whether lipoprotein metabolism contributes to disease progression.

Similar to observations in infectious disease, altered lipoprotein patterns and dysfunctional HDL have been identified in patients with SLE, RA, asthma, and psoriasis [91,92,93,94]. In addition to dysregulated lipid metabolism perpetuating the progression of immune disorders, affected patients often have an increased risk of cardiovascular disease [95,96]. Thus, therapies that target pathways of immune inflammation and lipid metabolism likely serve to reduce complications associated with both the primary disorder and related comorbidities. 

### 2.3. Summary: Role of Cholesterol in Immunity

**Figure 1:**
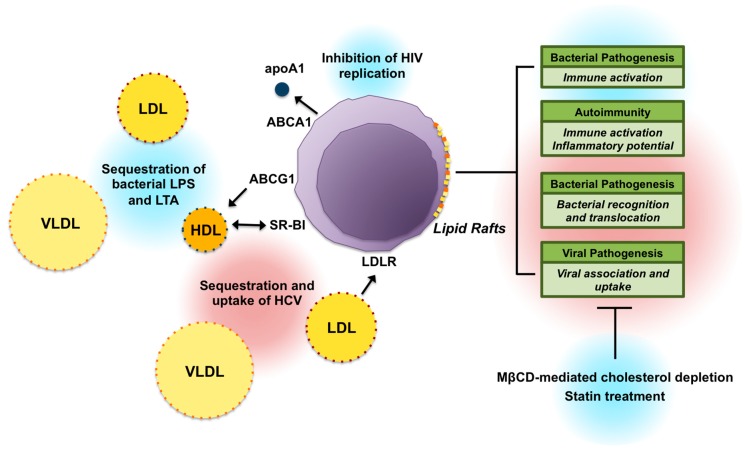
Role of cellular cholesterol and lipoprotein metabolism in immune function. Arrows indicate the direction of lipid efflux. Pathways highlighted in blue indicate protective mechanisms against disease, whereas pathways highlighted in red indicate mechanisms that promote disease pathogenesis. ABCA1: ATP-binding cassette transporter A 1; ABCG1: ATP-binding cassette transporter G 1; apoA1: apolipoprotein A 1; HCV: hepatitis C virus; HIV: human immunodeficiency virus; LDLR: low-density lipoprotein receptor; LPS: lipopolysaccharide; LTA: lipoteichoic acid; MβCD: methyl-β-cyclodextrin; SR-BI: scavenger receptor class B type I.

Taken together, research shows that lipid rafts and lipoprotein interactions are not only essential in the pathogenesis of infectious disease, but also in the compensatory immune response to ameliorate infection (Figure 1). Dysregulation of lipid pathways may additionally contribute to inappropriate leukocyte activation and inflammation in autoimmune disorders. [25]. While limited studies have investigated the direct relationship between lipid metabolism and immune function in humans, these compelling findings suggest that further research is warranted. As current evidence suggests the role of cholesterol metabolism varies across disease pathologies, it is further essential to elucidate the impact of lipid- and immune-modulating dietary components on risk and treatment of specific infectious diseases and autoimmune disorders.

## 3. Effects of Dietary Cholesterol and Egg Intake on Lipoprotein Metabolism and Immune Inflammation

Diets rich in cholesterol appear to have the capacity to regulate immune function through modulation of cellular cholesterol levels and lipoprotein metabolism [24]. The effects of dietary cholesterol and cholesterol-rich foods, specifically eggs, on plasma lipids have been reviewed by Blesso and Fernandez [97]; thus, the following sections focus on the effects of dietary cholesterol on immunomodulatory lipid pathways. In interpreting these findings, it is important to note that human studies evaluating the effects of dietary cholesterol often use whole eggs as the intervention treatment. Eggs are considered to be a rich source of dietary cholesterol, providing approximately 186 mg of cholesterol per large egg [97,98]. In addition to cholesterol, eggs contain a range of bioactive compounds that can impact lipid metabolism and inflammatory pathways, including glycerophospholipids, sphingolipids, proteins and peptides, and carotenoids [20]. Similarly, animal studies evaluating the effects of dietary cholesterol often utilize Western-style diets that contain excessively high levels of cholesterol, and are often rich in total fat and carbohydrates, such as sucrose [99,100]. Thus, for studies using eggs or Western-style diets as vehicles for dietary cholesterol, it is not possible to attribute observed effects solely to cholesterol within the context of a standard human diet.

### 3.1. Effects of Dietary Cholesterol and Egg Intake on Leukocyte Lipid Rafts and Cholesterol Metabolism

While few studies have assessed the effects of purified cholesterol on lipid raft formation and immune function, compelling evidence suggests that egg intake can modulate cholesterol metabolism in peripheral blood mononuclear cells (PBMCs), and that these effects are associated with changes in ABCA1 expression, inflammatory potential, and HDL function [24,101,102]. In a study by Andersen et al. [24], men and women classified with metabolic syndrome consumed 3 whole eggs per day vs. an egg white-based egg substitute for 12 weeks while following a moderate carbohydrate-restricted diet. Interestingly, there was a statistical trend towards reduced cholesterol content of peripheral blood mononuclear cells (*p* = 0.057) in subjects who consumed whole eggs, while PBMC cholesterol content did not change in participants who consumed the egg substitute. While there were no significant differences in total lipid raft content in either group over the 12-week intervention, changes in total PBMC cholesterol content positively correlated with changes in lipid raft content [24]. Interestingly, diets rich in carbohydrates, protein, and fat have been shown to differentially modulate cholesterol loading of PBMCs in healthy subjects [103].

Egg consumption has additionally been shown to modify the expression of genes involved in cellular cholesterol homeostasis in healthy, overweight, and metabolic syndrome populations. In metabolic syndrome subjects consuming 3 eggs per day during carbohydrate restriction, PBMC mRNA expression of ABCA1 and HMG-CoA reductase (HMGCR) increased, whereas ABCG1 and LDL-receptor (LDLR) mRNA expression was unchanged [24]. Sterol regulatory element-binding protein 2 (SREBP2)-mediated mRNA expression of HMGCR is known to increase as a compensatory response to cellular cholesterol levels, in line with the observed trend toward PBMC cholesterol reductions described above [24,104]. Conversely, in overweight men following a carbohydrate-restricted diet for 12 weeks, consumption of 3 eggs per day reduced PBMC mRNA expression of HMGCR and LDLR [105]. The discrepancy in HMGCR and LDLR expression patterns between studies may be related to variability of dietary cholesterol absorption, as insulin resistant individuals, such as those with metabolic syndrome, have a reduced absorption capacity compared to those who are insulin sensitive, which may impact cellular cholesterol loading [106,107]. Accordingly, PBMCs isolated from normal weight individuals are more enriched in cholesterol compared to PBMCs from overweight individuals [103].

In a crossover study conducted with healthy men and women, consumption of 3 eggs per day for 4 weeks reduced PBMC mRNA expression of HMGCR and SREBP2 compared to daily intake of a choline bitartrate supplement [108]. These findings suggest the whole egg components—such as cholesterol—impact markers of leukocyte cholesterol homeostasis beyond choline fractions. As described above, egg-induced reductions in HMGCR, LDLR, and SREBP2 mRNA expression observed in overweight and healthy adults may be due to compensatory mechanisms to suppress endogenous cholesterol synthesis and exogenous cholesterol uptake via LDL, presumably due to increased cellular cholesterol loading [104,105,108]. Accordingly, postprandial serum following egg intake in healthy men has been shown to increase free cholesterol content of J774 macrophages ex vivo [109]. However, these effects appear to be dose-dependent, as intake of 2 eggs per day for 4 weeks did not alter PBMC mRNA expression of cholesterol genes in healthy men and women [110]. Together, these studies suggest that egg intake alters leukocyte markers of cholesterol metabolism and lipid raft formation, and that dietary dose, cholesterol absorption capacity, and insulin resistance status may impact cellular responses.

### 3.2. Effects of Dietary Cholesterol and Egg Intake on Lipoprotein Metabolism and Function

Alterations in PBMC cholesterol gene expression from egg intake are likely impacted by interactions with lipoproteins. During weight maintenance, egg intake consistently increases LDL-cholesterol and HDL-cholesterol levels in approximately one-third of healthy populations that are considered to be “hyper-responders”, while often maintaining the LDL-cholesterol/ HDL-cholesterol ratio [111,112,113,114,115]. During weight loss, egg intake often increases HDL-cholesterol levels, without raising LDL-cholesterol [107,116,117]. Egg intake additionally increases LDL and HDL particle size, and has been shown to increase plasma levels of apoA1 and activity of the HDL-associated antioxidant paraoxonase 1 (PON1) [107,108,118,119,120]. Importantly, egg consumption during weight loss has been shown to increase the cholesterol-accepting capacity of serum and reduce pro-inflammatory SAA in metabolic syndrome subjects, which was associated with a trend toward reduced PBMC cholesterol loading [24,101,102]. Conversely, consumption of 4 eggs per day for 4 weeks increased SAA levels in healthy adults during weight maintenance, which may lead to a reduced efflux capacity of HDL [121,122]. Investigation of leukocyte interactions with specific lipoprotein classes is further warranted, as surprisingly, plasma LDL levels and ex vivo culture with lipoprotein-rich vs. lipoprotein-deficient media did not impact cholesterol loading of PBMCs from healthy volunteers [103]. Taken together, the variable effects of egg intake on lipoprotein profiles observed between healthy vs. insulin resistant populations reflect differences in PBMC cholesterol loading and gene expression, suggesting that lipoprotein profiles and functionality may differentially drive cholesterol metabolism in leukocytes based on metabolic status.

### 3.3. Effects of Dietary Cholesterol and Egg Intake on Leukocyte Inflammation

Given the relationship between cellular cholesterol homeostasis and immune activation, it is further likely that egg-induced changes to cholesterol metabolism and lipoprotein profiles impact inflammatory leukocyte responses. In metabolic syndrome subjects consuming 3 eggs per day during moderate carbohydrate restriction, PBMC mRNA expression of TLR4 increased after 12 weeks; however, there was no change in LPS-induced TNFα and IL-1β secretion in ex vivo PBMCs. Conversely, LPS-induced TNFα and IL-1β secretion increased over 12 weeks in ex vivo PBMCs derived from subjects consuming an egg white-based substitute, suggesting that yolk-derived components may have blunted responsiveness to bacterial LPS [24]. Plasma levels of TNFα were also reduced after 12 weeks in the whole egg group [102]. Egg intake has additionally been shown to reduce TNFα and aspartate aminotransferase (AST) in type 2 diabetic patients [123], as well as decrease CRP and increase anti-inflammatory adiponectin in overweight men during weight loss [124]. Conflicting results have been reported in healthy adults, where egg intake has been shown to increase CRP [121] and postprandial IL-6 [125], whereas fasting AST and alanine aminotransferase (ALT) are not affected [126]. Discrepancies in inflammatory responses across studies may be attributable to the degree of weight loss, insulin sensitivity, cholesterol absorption, and modulation of lipoprotein profiles and leukocyte cholesterol metabolism, as described above [24,101,106,109,127].

In animal models, Western-style diets have additionally been shown to promote transcriptional and epigenomic reprogramming of myeloid progenitor cells, contributing to enhanced proliferative and proinflammatory responses [128]. Cholesterol feeding has further been shown to dose-dependently increase FoxP3+ T regulatory (Treg) cell numbers in the spleen and thymus of C57BL/6 mice, with Treg cells derived from animals fed cholesterol-containing diets having a greater capacity to suppress proliferation of CD4+ responder T cells. Despite the increase in anti-inflammatory Treg cell populations, cholesterol feeding increased TCR responsiveness and proliferation in CD4+ T cells, which may outweigh the suppressive effects of Treg cells and drive proinflammatory responses in these models [129].

### 3.4. Summary: Effects of Dietary Cholesterol and Egg Intake on Lipid Metabolism and Immune Inflammation

Taken together, it appears that consumption of cholesterol-rich eggs and Western-style dietary patterns impact leukocyte cholesterol metabolism and inflammatory potential, and that cellular interactions with lipoproteins may contribute to these effects. Further, the effects of egg intake on lipid metabolism and immune inflammation appear to vary across human populations and metabolic status. These findings have important implications for the pathophysiology and management of infectious disease and autoimmune disorders, suggesting that immunomodulatory lipid pathways may serve as a therapeutic target for dietary interventions that modify cholesterol intake, or provide other dietary bioactives that impact lipid metabolism. 

## 4. Dietary Cholesterol Effects in Infectious Disease

Cholesterol-rich foods and dietary patterns have the potential to modulate cellular cholesterol metabolism and lipoprotein function. Given the role of cholesterol-rich lipid rafts and lipoprotein interactions in mediating infection by numerous bacterial and viral pathogens [38,39], understanding the impact of dietary cholesterol on these pathways may influence prevention, assessment, and treatment of disease. Interestingly, while diets rich in cholesterol may exasperate and increase the risk of bacterial pulmonary infection in animal models and human populations, respectively, cholesterol intake during active infection may promote tissue-specific pathogen clearance and clinical outcomes (Table 1). Studies in animals and humans further show that dietary cholesterol may exasperate viral infections; however, responses may be gender-specific (Table 2). The following sections compare findings from research studies evaluating the effects of dietary cholesterol on the pathogenesis of infectious diseases. 

### 4.1. Tuberculosis

Tuberculosis is one of the leading causes of death from infectious disease worldwide [139]. Tuberculosis can further lead to, and be complicated by, malnutrition and nutrient deficiencies [140]. Research on nutritional support for tuberculosis has been inconsistent, leading to a lack of nutritional guidelines and recommendations for tuberculosis treatment [140]. Lipid raft formation is essential for *Mycobacterium tuberculosis* internalization and survival in host cells [141], whereas serum lipid levels are reduced and negatively associated with clinical radiological and smear positivity measures on pulmonary tuberculosis patients [142,143]. Thus, cellular cholesterol enrichment may promote *M. tuberculosis* infection, whereas systemic cholesterol depletion may contribute to disease progression.

Accordingly, conflicting effects of dietary cholesterol on tuberculosis pathology have been reported (Table 1). In apolipopotein E (apoE)-deficient mice, high cholesterol feeding exasperated *M. tuberculosis* infection, as evidenced by reduced T helper 1 (Th1)-mediated immune responses, increased bacterial burden and lung tissue inflammation, and early onset mortality [130]. Schafer et al. [131] additionally found that high cholesterol (1.25% cholesterol) feeding resulted in a greater bacterial burden of *M. tuberculosis* H37Rv in both wild type and SB-BI knockout mice, as compared to mice consuming a low cholesterol (0.15%) diet. However, pulmonary histopathology and cytokine expression did not differ between diet treatment groups [131]. In 63,257 men and women participating in the Singapore Chinese Health Study, dietary cholesterol intake was dose-dependently associated with risk of active tuberculosis, where individuals consuming the greatest amount of cholesterol had the greatest risk of tuberculosis [132]. Conversely, patients who consumed a cholesterol-rich diet (800 mg/day) while undergoing inpatient treatment for active pulmonary tuberculosis had faster reductions in positive sputum cultures and sputum production, as compared to patients receiving a low cholesterol diet (250 mg/day), suggesting a more rapid clearance of active infections [133]. Together, these findings suggest that habitually high intakes of dietary cholesterol may promote *M. tuberculosis* infection, whereas supplementation during active infection may promote pathogen clearance and recovery. 

### 4.2. Pneumonia

In addition to tuberculosis, dietary cholesterol appears to impact the pathogenesis of other types of pulmonary infections, including pneumonia. *Klebsiella pneumoniae*—a pneumonia-causing Gram-negative pathogen—additionally utilizes host lipid rafts for infection, whereas depletion of host cell cholesterol by MβCD and LXR activation impairs *K. pneumonia* internalization and subsequent host defenses [144,145]. These findings suggest that cholesterol enrichment may promote *K. pneumonia* clearance.

Animal studies have demonstrated that high cholesterol feeding modifies the lipid composition of pulmonary tissue. In mice fed a high cholesterol (2%) diet, surface-active film from pulmonary surfactant was found to be enriched in cholesterol, which was associated with reduced surface-active film stability [146]. High intake of dietary cholesterol has also been associated with modification of the glycerophospholipid composition of lung surfactant, increasing phosphatidylglycerol while decreasing phosphatidylethanolamine [147]. Interestingly, C57BL/6 mice fed a cholesterol-rich (1.25%) Western diet exhibited compromised bacterial clearance from pulmonary tissues, despite more rapid clearance of pathogens from the bloodstream [134]. High cholesterol feeding additionally increased serum levels of pro-inflammatory IL-17 and its target protein, macrophage-inflammatory protein 2 (MIP-2), while suppressing LPS-induced pulmonary TNFα, MIP-2, and nuclear factor ĸ B (NF-ĸB) p65 subunit activation. Lung-derived polymorphonuclear (PMN) cell numbers and chemotaxis were similarly reduced by high cholesterol feeding, while extra-pulmonary bacterial burden in spleen and liver were significantly reduced. Overall, survival rates did not statistically differ between high- and low-cholesterol groups, although the authors note that the high cholesterol group tended to have greater survival [134]. Together, these findings suggest that cholesterol dose-dependently modulates pathways of *K. pneumonia* in a tissue-specific manner, which may ultimately impact disease outcomes.

The effects of dietary cholesterol on clinical outcomes of pneumonia have additionally been investigated in humans. In a study by Wang and Hong [135], pneumonia patients were supplemented with an additional 600 mg of cholesterol from egg yolks per day for 10 days. Interestingly, patients receiving egg yolks exhibited significantly lower high-sensitivity CRP (hsCRP) levels compared to the control group, in addition to lower plasma IL-6. Egg yolk supplementation also improved ratings for simplified acute physiology score II (SAPS II) and Subjective Global Assessment, which is indicative of reduced disease severity and improved nutritional status [135]. While total cholesterol levels increased following egg yolk intake, markers of HDL function and leukocyte cholesterol metabolism were not assessed. Given that these parameters are modified by egg intake in other studies, it is possible that these factors played a role in the immune response [24,101,102]. Thus, while excess dietary cholesterol may exasperate pulmonary dysfunction during *K. pneumonia* infection in animal models [134], systemic effects and egg consumption in humans may lead to clinical benefits overall (Table 1).

### 4.3. Hepatitis C Virus

In addition to infectious diseases affecting pulmonary tissues, dietary cholesterol may further impact mechanisms of viral infection by hepatic pathogens (Table 2). As described above, HCV infection can be mediated by lipoprotein-dependent mechanisms, including viral transport by lipoproteins and cellular uptake by the LDL-receptor and SR-BI [74,75]. Using stable isotope methods, Lambert et al. [148] demonstrated that individuals with HCV infection displayed increased rates of fasting whole body and hepatic lipogenesis, as evidenced by greater levels of de novo fatty acids in plasma triacylglycerols (TAG) and VLDL-TAG, respectively. Interestingly, de novo synthesis of free cholesterol and cholesteryl esters was significantly reduced in HCV patients [148]. Additional studies suggest that while HCV infection induces expression of genes involved in endogenous cholesterol synthesis, chemical intermediates may be diverted into non-cholesterol pathways to support viral replication [148,149,150]. Accordingly, Corey et al. [151] demonstrated that patients with HCV infection exhibit decreased total and LDL-cholesterol levels, which are normalized following successful HCV treatment.

Given the disruptions in hepatic metabolism, HCV-infected individuals are at greater risk of developing hepatic fibrosis, cirrhosis, and carcinoma with disease progression [152,153]. Consequently, HCV patients may be more susceptible to inflammatory damage from excess dietary cholesterol. Dietary cholesterol has been shown to promote hepatic inflammation, steatosis, and fibrosis in animal models, whereas these effects may be exasperated by concomitant high-fat and high-sucrose feeding in Western-style diets [154,155,156,157]. In 608 patients with chronic HCV infection, individuals with greater intake of dietary cholesterol (224–310 mg/day and >310 mg/day) had increased risk for disease progression toward fibrosis and/or cirrhosis, as defined by increases in histological Ishak fibrosis scores in liver biopsies [136]. Interestingly, Yu et al. [137] further demonstrated that dietary cholesterol intake was dose-dependently associated with increased risk of liver-related death and transplantation in women with chronic HCV infection and advanced fibrosis or compensated cirrhosis, whereas a relationship between dietary cholesterol and liver disease progression was not observed in men. Female mice have previously been shown to exhibit greater efficiency of cholesterol absorption, increased hepatic cholesterol loading, and larger increases in bile acid pools in response to dietary cholesterol, suggesting that females may be more sensitive to exogenous cholesterol intake [158].

In a pilot intervention trial, subjects with chronic HCV infection following a normocaloric low-cholesterol (185 mg/day) diet for 30 days had an increased percentage of anti-inflammatory Treg lymphocytes as compared to baseline, in addition to reduced pro-inflammatory T helper 17 (Th17) lymphocytes, and lower serum levels of IL-17, IL-22, transforming growth factor β (TGFβ), and hyaluronic acid [26]—a biomarker that positively correlates with hepatic fibrosis [159]. Elevated Th17-mediated responses have been implicated in promoting hepatic inflammation, steatosis, and hepatocellular carcinoma [160,161]. PBMC mRNA expression of LXRα, LXRβ, sterol regulatory binding protein 1c (SREBP-1c), and ABCA1 additionally increased following the low cholesterol diet [26], which may represent a compensatory mechanism in response to changes in cellular cholesterol levels [162]. Similar to what has been observed with cholesterol-rich egg feeding [24], these findings suggest that restricting dietary cholesterol modulates blood leukocyte lipid metabolism and inflammatory profiles [26]. Given that Th17 lymphocytes have been implicated in numerous inflammatory diseases and immune disorders [163], the role of dietary cholesterol in regulating this T lymphocyte population warrants further investigation.

### 4.4. Human Immunodeficiency Virus (HIV)

HIV pathogenesis similarly involves HDL and ABCA1 pathways, where cholesterol depletion and efflux appear to confer therapeutic effects [69,71,72,73]. Egg intake has previously been shown to increase PBMC expression of ABCA1 and the cholesterol-accepting capacity of serum [24,101], as well as modify mRNA expression of genes involved in cholesterol homeostasis [24,105,108], suggesting that cholesterol-rich foods may modulate mechanisms of HIV infection. While research on dietary cholesterol intake and HIV pathogenesis is limited, Mansfield et al. [138] examined the effects of a high cholesterol (1%)/high fat (~40% of energy) diet on simian immunodeficiency virus (SIV) progression to acquired immunodeficiency syndrome (AIDS) in infected macaques primates. Interestingly, animals fed the high cholesterol/high fat diet exhibited greater peak viral loads compared to animals fed a control diet. High cholesterol/high fat diet feeding additionally resulted in faster disease progression and risk of SIV-related death, corresponding to elevated IL-18 levels in plasma, as well as greater incidence of concomitant infections and body wasting [138]. Elevated IL-18 levels have additionally been observed in HIV-infected patients, and may be involved in promoting viral replication, lipodystrophy, and Th2 lymphocyte responses that accelerate disease progression [138,164,165,166]. Further clinical research is warranted to determine whether cholesterol-rich foods or dietary patterns impact lymphocyte cholesterol loading and efflux in HIV within the context of initial infection and treatment in humans. 

## 5. Dietary Cholesterol Effects in Autoimmune Disease

In addition to infectious diseases, dietary cholesterol has the potential to impact risk, severity, and treatment of chronic autoimmune disorders. Potential targets of exogenous cholesterol may include regulation of leukocyte cholesterol metabolism and lipid raft formation, alteration of lipoprotein profiles and immunomodulatory functions, and modification of systemic tissue stress and inflammation [24,101,102,108,167]. Interestingly, dietary cholesterol appears to exasperate autoimmune inflammation and risk, whereas cholesterol restriction reduces inflammation and improves clinical outcomes (Table 3). An exception to this trend is observed in models of demyelinating autoimmune disorders, where cholesterol supplementation improves central nervous system (CNS) integrity and inflammation [168]. The following sections highlight research evaluating the effects of dietary cholesterol on the pathophysiology of autoimmune conditions in animal models and human populations. 

### 5.1. Asthma

Asthma is a chronic inflammatory disorder with a potential autoimmune etiology that affects the lower respiratory tract [175,176]. High cholesterol diets lead to enrichment of pulmonary surfactant cholesterol and phosphatidylglycerol levels, which reduces surface-active film stability and may lead to altered pulmonary leukocyte activity and immunity to pulmonary pathogens [133,134,146,147]. In an ovalbumin-sensitized C57BL/6 mouse model for asthma, diets rich in cholesterol (1% and 2%) dose-dependently induced greater bronchoaveolar inflammation, as evidenced by increased levels of eosinophils, IL-5, and cysteinyl leukotrienes compared to control fed mice (0.02% cholesterol) [27]. In a similar study, Yeh and Huang [169] demonstrated that ovalbumin-sensitized mice fed high cholesterol (2%) diets exhibited increased pro-inflammatory IL-5, prostaglandin E 2 (PGE2), monocyte chemoattractant protein 1 (MCP-1) and eosinophil numbers in bronchoalveolar lavage fluid, in addition to greater IL-4 and interferon γ (IFNy) production by lung-derived lymphocytes. Dietary cholesterol additionally reduced levels of IL-12, which has been shown to have both pro-inflammatory effects and therapeutic potential in asthma [177,178]. Interestingly, the addition of pravastatin treatment reduced pulmonary inflammation, with the exception of increasing IL-12 [169]. In a study evaluating 156,035 Australian adults from The 45 and Up Study, Rosenkranz [170] et al. demonstrated that men and women who consumed diets rich in meats, poultry, and seafood had greater odds of receiving a diagnosis for asthma/hayfever. These findings suggest that cholesterol-containing animal foods may contribute to the development of asthma or hayfever. However, these associations may be gender- and food source-specific, as cheese was found to be positively associated with asthma and hayfever in men, whereas dietary patterns consisting of cheese and whole grain bread were found to be protective in women [170]. Additional observational studies have found neutral or positive associations between Western-style diet patterns and asthma (Table 3) [179,180]. Further intervention studies are needed to elucidate the impact of dietary cholesterol on asthma in humans, and determine whether variability in lipid metabolism plays a role in the controversial findings observed between genders. 

### 5.2. Rheumatoid Arthritis

RA is a chronic autoimmune disorder characterized by severe joint inflammation and damage. Activated T lymphocytes are found within synovial joint fluid from RA patients, yet they exhibit impaired TCR responsiveness and proliferative capacity [87,88]. Proinflammatory HDL and reduced HDL-mediated cholesterol efflux has additionally been observed in RA patients [94,181,182,183], whereas RA treatment improves markers of HDL function [184] and increases PBMC mRNA ABCA1 expression [185]. Increases in HDL-cholesterol have additionally been associated with improvements in radiographic hand osteoarthritis [186]. However, it is unclear whether cholesterol metabolism or lipid raft formation is modified within activated T cell populations, or whether cholesterol-rich diets can directly modulate HDL dysfunction and T lymphocyte activity in joint tissues of RA patients [24,101]. Interestingly, dietary regimens that are low in cholesterol or cholesterol-free—including medically supervised fasting (7–10 days), vegan diets, and lactovegetarian diets—have been shown to reduce inflammation and improve clinical measures of RA [187]. Hafström et al. [171] demonstrated that a greater percentage of RA patients following a gluten-free vegan diet for at least nine months exhibited clinical improvement according to the American College of Rheumatology 20 (ACR20) criteria, as compared to patients consuming a non-vegan diet. Patients following a 4-week, very low-fat diet (10%) vegan diet additionally experienced improved RA symptoms, including a reduction in pain, joint swelling, and joint mobility [172]. Vegan and vegetarian diets have further been shown to reduce total cholesterol and LDL-cholesterol levels in RA patients [188,189], as well as reduce leukocyte counts and pro-inflammatory CRP [173,174]. Conversely, high cholesterol diets have been shown to exasperate joint inflammation and osteoarthritis development in APOE*3Leiden.CETP mice, potentially due to cholesterol-induced inflammation and joint cartilage degradation [28,190,191]. These findings suggest that dietary cholesterol restriction improves RA outcomes, yet further research is warranted to elucidate the mechanisms by which this occurs. 

### 5.3. Systemic Lupus Erythematous (SLE)

Systemic Lupus Erythematous (SLE) is classified as a systemic autoimmune disease, where autoreactive T and B lymphocytes mediate the production of autoantibodies against nuclear components. Clinical consequences of SLE include multiorgan tissue inflammation and injury [192]. SLE patients often present with dyslipidemia and dysfunctional HDL, which lead to an increased risk of cardiovascular disease, and potentially exasperate hyper-responsive, proinflammatory T lymphocyte responses [80,81,92,95,193,194]. Dietary recommendations for SLE patients often include restrictions of dietary cholesterol as a means to address dyslipidemia and cardiovascular disease risk; however, few studies have evaluated the direct effects of dietary cholesterol on autoimmune inflammation and clinical outcomes of SLE [195,196,197]. In ApoE/LXRβ-deficient mice, feeding of a cholesterol-containing (0.21%) Western diet resulted in the development of a lupus-like disease [100]. Western diet feeding increased cholesterol accumulation in the spleen and lymph nodes, while enhancing T cell priming, B cell expansion, and production of autoantibodies. Conversely, B cell expansion and autoantibody production was suppressed by enhancing mechanisms of HDL-mediated cholesterol efflux [100]. While additional studies are required to fully elucidate the effects of dietary cholesterol SLE-related immune dysfunction in humans, current evidence suggests that excess cholesterol loading may be involved in the pathogenesis of SLE-like autoimmunity, whereas dietary cholesterol restriction and lipid efflux-promoting therapeutics may support clinical management of SLE complications.

### 5.4. Multiple Sclerosis

Multiple sclerosis (MS) is a proinflammatory autoimmune disorder that causes demyelinization and damage of CNS cells [198]. While dietary cholesterol may exasperate inflammation in certain autoimmune conditions, studies have found that exogenous cholesterol may improve inflammation and degenerative pathways associated with MS. Berghoff et al. [168] demonstrated that high cholesterol (5%) feeding reduced immune cell infiltration into the spinal cords of experimental autoimmune encephalomyelitis (EAE) murine models of MS, in addition to reducing proinflammatory spinal cord mRNA expression of TNFα, IL-17, IFNγ, granulocyte-monocyte colony-stimulating factor (GM-CSF), and major histocampatability complex II (MHCII). However, dietary cholesterol did not improve overall clinical scoring of CNS lesions and disease pathology in EAE mice. Interestingly, high cholesterol (2%) feeding promoted remyelinaization in cuprizone- and lysolecithin-induced mouse models of MS. Dietary cholesterol additionally increased oligodendrocyte precursor cell proliferation, oligodendrocyte differentiation, and improved motor function in the cuprizone-treated mice, while it was unable to prevent initial demyelinization [168]. These findings may be translatable to other demyelinating disorders, as cholesterol has been shown to be a rate-limiting factor in myelin production in CNS cells [199]. Accordingly, Saher et al. [200] found that dietary cholesterol improved myelination, inflammation, and motor control in a mouse model of Pelizaeus-Merzbacher disease—a fatal leukodystrophy condition of genetic etiology that currently lacks effective treatment options. Thus, dietary cholesterol may serve as a safe and effective therapeutic strategy to address demyelinating disorders. 

## 6. Conclusions

**Figure 2:**
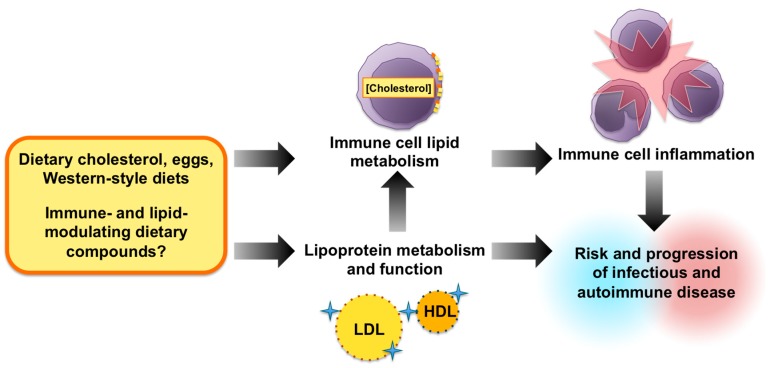
Proposed relationship between diet, lipid metabolism, and immune function in the pathophysiology of infectious and autoimmune disease.

Overall, it is clear that cholesterol metabolism plays an important, yet complex and controversial role in the regulation of immunity and inflammatory disease risk (Figure 2). Consumption of dietary cholesterol, specifically coming from eggs, has been shown to modulate leukocyte cholesterol metabolism, lipid raft dynamics, and inflammatory potential, while additionally modulating lipoprotein profiles and markers of HDL function [24,101]. Interestingly, evidence from human studies suggests that these pathways are affected by cholesterol dose and metabolic status, suggesting that efficiency of dietary cholesterol absorption, the presence of insulin resistance, and weight loss can impact the immunomodulatory response to exogenous cholesterol [105,106,108,110]. Research from animal and human studies further demonstrates that cholesterol-rich dietary patterns differentially impact pathophysiology and clinical outcomes of distinct infectious diseases by various bacterial and viral pathogens, and that dietary cholesterol may either exasperate or mitigate autoimmune dysfunction [26,100,133,135,168,169]. However, further human studies and clinical trials are required to determine whether altered leukocyte cholesterol metabolism, lipoprotein profiles, or metabolic status underlie the variable effects of cholesterol-rich diet intake on infectious and autoimmune disease pathophysiology. It is additionally essential to elucidate the contributions of confounding dietary bioactives in these pathways, and evaluate whether other functional foods known to modulate lipid metabolism can impact immunity via similar mechanisms. Together, the findings presented in this review highlight a significant role for dietary cholesterol in human health beyond the scope of cardiovascular disease.

## Figures and Tables

**Table 1:** Comparative effects of dietary cholesterol on infection by bacterial pathogens.

Experimental Model/Population	Dietary Conditions	Effect	Reference
**Tuberculosis**			
ApoE-deficient mice	High cholesterol (1.25%) diet	↓ Th1 response ↑ bacterial burden, lung inflammation, early onset mortality	[130]
Wild type and SR-BI knockout mice	High cholesterol (1.25%) diet	↑ bacterial burden ↔ pulmonary histopathology, cytokine expression	[131]
Men and women in the Singapore Chinese Health Study, *n* = 63,257	Observational study	↑ increased risk of active TB	[132]
Active pulmonary TB patients, *n* = 21	Cholesterol-rich diet (800 mg/day) vs. normocholesterolemic diet (250 mg/day)	↓ positive sputum cultures, sputum production	[133]
**Pneumonia**			
C57BL/6 mice	Cholesterol-rich (1.25%) Western Diet	↓ pulmonary bacterial clearance, pulmonary PMN numbers and chemotaxis, LPS-induced pulmonary TNFα, MIP-2, NF-ĸB p65 subunit activation ↑ rate of clearance of pathogens from blood, serum TNFα, MIP-2 ↓ bacterial burden in spleen and liver ↔ survival rates	[134]
Pneumonia patients, *n* = 47	600 mg/day from egg yolks for 10 days	↓ plasma CRP, IL-6 ↑ SAPSII and SGA scores	[135]

↓: Decreased; ↑: increased; ↔: no change or difference between groups. Abbreviations: apoE: apolipoprotein E; CRP: C-reactive protein; IL-6: interleukin 6; LPS: lipopolysaccharide; MIP-2: macrophage-inflammatory protein 2; NF-ĸB: nuclear factor ĸ B; PMN: polymorphonuclear cells; SAPSII: Simplified Acute Physiology Score II; SGA: Subjective Global Assessment; SR-BI: scavenger receptor class B type I; TB: tuberculosis; Th1: T helper 1 lymphocytes; TNFα: tumor necrosis factor α.

**Table 2:** Effects of dietary cholesterol on the pathophysiology of viral infections.

Experimental Model/Population	Dietary Conditions	Effect	Reference
**Hepatitis C Virus**			
Chronic HCV patients, *n* = 608	Observational study: 224–310 mg cholesterol/day; >310 mg cholesterol/day	↑ risk for fibrosis and/or cirrhosis	[136]
Chronic HCV patients from the HALT-C Trial with advanced fibrosis or compensated cirrhosis, *n* = 657	Observational study	↑ risk of liver-related death and transplantation in women ↔ relationship between dietary cholesterol and disease progression in men	[137]
Chronic HCV patients, *n* = 30	Normocaloric, low-cholesterol (185 mg/day) diet for 30 days	↑ % Treg cells, PBMC mRNA expression of LXRα, LXRβ, SREBP-1c, ABCA1 ↓ % Th17 cells, serum IL-17, IL-22, TGFβ, HA	[26]
**HIV/AIDS**			
SIV-infected macaque primates	High-fat (40% of energy)/high-cholesterol (1%) diet	↑ peak viral loads, rate of disease progression, plasma IL-18, incidence of co-infections, body wasting, risk of SIV-related death	[138]

↓: Decreased; ↑: increased; ↔: no change or difference between groups. Abbreviations: ABCA1: ATP-binding cassette transporter A 1; AIDS: acquired immunodeficiency syndrome; apoE: apolipoprotein E; HA: hyaluronic acid; HCV: hepatitis C virus; HIV: human immunodeficiency virus; IL-17: interleukin 17; IL-18: interleukin 18; IL-22: interleukin 22; LXRα: liver X receptor α; LXRβ: liver X receptor β; PBMC: peripheral blood mononuclear cell; SIV: simian immunodeficiency virus; SREBP-1c: sterol regulatory binding protein 1 c; TB: tuberculosis; TGFβ: transforming growth factor β; Th17: T helper 17 lymphocytes; TNFα: tumor necrosis factor α; Treg: T regulatory lymphocytes.

**Table 3:** Differential effects of dietary cholesterol on the pathophysiology of autoimmune disorders.

Experimental Model/Population	Dietary Conditions	Effect	Reference
**Asthma**			
Ovalbumin-sensitized C57BL/6 mice	Cholesterol-rich (1% and 2%) diets	↑ bronchoaveolar inflammation, eosinophil numbers, IL-5, cysteinyl leukotrienes	[27]
Ovalbumin-sensitized C57BL/6 mice	Cholesterol-rich (2%) diet	↑ IL-5, PGE2, MCP-1, eosinophils numbers in bronchoalveolar lavage fluid ↑ IL-4, IFNγ production by pulmonary lymphocytes ↓ IL-12	[169]
Men and women from The 45 and Up Study, *n* = 156,035	Diets rich in meats, poultry, and seafood	↑ odds of asthma/hayfever diagnosis	[170]
Men and women from The 45 and Up Study, *n* = 156,035	Diets rich in cheese	↑ odds of asthma/hayfever diagnosis in men ↓ odds of asthma/hayfever diagnosis in women	[170]
**Rheumatoid arthritis**			
RA patients, *n* = 47	Gluten-free, vegan diet for nine months vs. non-vegan diet	↑ clinical improvement by ACR20 criteria	[171]
RA patients, *n* = 24	Very low-fat (10% of energy) vegan diet	↓ joint pain and swelling ↑ joint mobility	[172]
RA patients, *n* = 53	7–10 day fast → gluten-free vegan diet vs. standard non-vegan diet	↓ number of tender and swollen joints, pain score, duration of morning stiffness, white blood cell count, CRP ↑ grip strength	[173]
RA patients, 53	7–10 day fast → gluten-free vegan diet → vegetarian diet vs. a standard non-vegan diet	↓ leukocyte and platelet counts, total IgG and IgM rheumatoid factor, calprotectin, C3 and C4 complement proteins	[174]
APOE*3Leiden.CETP mice	Cholesterol-rich (0.1% and 0.3%) Western diets	↑ joint inflammation, osteoarthritis development	[28]
**Systemic Lupus Erythematous**			
ApoE/LXRβ-deficient mice	Cholesterol-rich (0.21%) Western diet	↑ cholesterol accumulation in spleen and lymph nodes, T cell priming, B cell expansion, autoantibody production * Note: HDL-mediated efflux suppressed diet-induced B cell expansion and autoantibody production	[100]
**Multiple sclerosis**			
EAE mouse model of MS	High cholesterol (5%) diet	↓ spinal immune cell infiltration, mRNA expression of TNFα, IL-17, IFNγ, GM-CSF, MHCII ↔ clinical scoring of CNS lesions and disease pathology	[168]
Cuprizone-induced mouse model of MS	High cholesterol (2%) diet	↔ demyelinization ↑ remyelinization, oligodendrocyte precursor cell proliferation, oligodendrocyte differentiation, motor function	[168]
Lysolechithin-induced mouse model of MS	High cholesterol (2%) diet	↑ remyelinization	[168]

↓: Decreased; ↑: increased; ↔: no change or difference between experimental and control groups. Abbreviations: apoE: apolipoprotein E; CETP: cholesteryl ester transfer protein; CRP: C-reactive protein; EAE: experimental autoimmune encephalomyelitis; GM-CSF: granulocyte-monocyte colony stimulating factor; IgG: immunoglobulin G; IgM: immunoglobulin M; IL-12: interleukin 12; IL-17: interleukin 17; IL-4: interleukin 4; IL-5: interleukin 5; IFNγ: interferon γ; LXRβ: liver X receptor β; MCP-1: monocyte chemoattractant protein 1; MS: multiple sclerosis; PGE2: prostaglandin E2; RA: rheumatoid arthritis; TNFα: tumor necrosis factor α.
